# A Novel Fast-Setting Strontium-Containing Hydroxyapatite Bone Cement With a Simple Binary Powder System

**DOI:** 10.3389/fbioe.2021.643557

**Published:** 2021-03-18

**Authors:** Lijuan Sun, Tongyang Li, Sen Yu, Mengmeng Mao, Dagang Guo

**Affiliations:** State Key Laboratory for Mechanical Behavior of Materials, School of Material Science and Engineering, Xi’an Jiaotong University, Xi’an, China

**Keywords:** hydration reaction, strontium, physicochemical property, cytocompatibility, calcium phosphate bone cement

## Abstract

In recent years, strontium-substituted calcium phosphate bone cement (Sr-CPC) has attracted more and more attentions in the field of bone tissue repair due to its comprehensive advantages of both traditional CPC and Sr ions. In this study, a crucial Sr-containing α-Ca_3__–_*_*x*_*Sr*_*x*_*(PO_4_)_2_ salt has been synthesized using a simplified one-step method at lower synthesis temperature. A novel Sr-CPC has been developed based on the simple binary Sr-containing α-Ca_3__–_*_*x*_*Sr*_*x*_*(PO_4_)_2_/Ca_4_(PO_4_)_2_O cement powder. The physicochemical properties and hydration mechanism of this Sr-CPC at various Sr contents were intensively investigated. The setting product of this Sr-CPC after a set for 72 h is a single-phase Sr-containing hydroxyapatite, and its compressive strength slightly decreased and its setting time extended with the increase of Sr content. The hydration process included the initial formation of the medium product CaHPO_4_⋅2H_2_O (30 min∼1 h), the following complete hydration of Ca_4_(PO_4_)_2_O and the initially formed CaHPO_4_⋅2H_2_O (2∼6 h), and the final self-setting of α-Ca_3__–_*_*x*_*Sr*_*x*_*(PO_4_)_2_ (6 h∼). The compressive strength of Sr-CPC, which was closely related to the transformation rate of Sr-containing hydroxyapatite, tended to increase with the extension of hydration time. In addition, Sr-CPC possessed favorable cytocompatibility and the effect of Sr ions on cytocompatibility of Sr-CPC was not obvious at low Sr contents. The present study suggests α-Ca_3__–_*_*x*_*Sr*_*x*_*(PO_4_)_2_ is a kind of vital Sr-containing salt source which is useful to develop some novel Sr-containing biomaterials. In addition, the new Sr-containing cement system based on this simple binary α-Ca_3__–_*_*x*_*Sr*_*x*_*(PO_4_)_2_/Ca_4_(PO_4_)_2_O cement powder displayed an attractive clinical application potential in orthopedics.

## Introduction

In recent years, strontium-substituted calcium phosphate bone cement (Sr-CPC) attracted increasing concerns in fields of bone tissue repair and spine fixing. Except that this new biomaterial possesses a lot of same merits with conventional Sr-free CPC, Sr element exhibits many favorable roles in human bone, including enhancing bone strength (Guo et al., 2005a), accelerating new bone tissue formation, curing osteoporosis (Chapuy and Meunier, 1995; Ozeki et al., 2013), treating bone cancer and alleviating bone pain (Roberts et al., 2002), etc. Moreover, Sr is a kind of trace elements in human body, sharing the same physiological pathway as Ca and can be deposited into the mineral structure of bone, especially into the regions of high metabolic turnover (Blake et al., 1986). It has been reported that the partial replacement of Ca^2+^ ions in HAP by equal molar ratio of Sr^2+^ ions significantly increases both the mechanical properties (Chen and Fu, 2001) and the degradation rate (Christoffersen et al., 1997; Guo et al., 2008) of HAP. Therefore, as a kind of novel injectable and radiopaque hydraulic cement, Sr-CPCs have a more attractive clinical application potential than the conventional Sr-free CPC.

Up to now, dozens of Sr-CPCs (Li et al., 2000; Lerous and Lacout, 2001; Cheung et al., 2005; Guo et al., 2005a; Saint-Jean et al., 2005; Wang et al., 2007; Alkhraisat et al., 2008a; Panzavolta et al., 2008; Yu et al., 2009; Pina et al., 2010; Romieu et al., 2010; Kuang et al., 2012; Tadier et al., 2012; Baier et al., 2013; Schumacher et al., 2013; Tao et al., 2013; López et al., 2014; Zhou et al., 2015; D’Onofrio et al., 2016; Singh et al., 2016; Huang et al., 2017; Jayasree et al., 2017; Shi et al., 2017; Taha et al., 2017; Zhu et al., 2017; Lode et al., 2018; No et al., 2019; Wu et al., 2019) with different cement powder compositions or different cement liquid formulations have been developed (see Table 1). As is well known, the Sr^2+^ ions could be introduced into the final cement product *via* cement powder or cement liquid. The major Sr-containing salts used in the cement powder composition include the pre-treated Sr-HAP (Li et al., 2000; Cheung et al., 2005), Sr-α-TCP (Saint-Jean et al., 2005; Pina et al., 2010), SrCl_2_ (Alkhraisat et al., 2008a; Panzavolta et al., 2008; Tadier et al., 2012; López et al., 2014), DSPA (Guo et al., 2005a; Kuang et al., 2012; Baier et al., 2013), Sr-ACP (Yu et al., 2009), SC (Saint-Jean et al., 2005; Wang et al., 2007; Romieu et al., 2010; Tadier et al., 2012; Schumacher et al., 2013; Huang et al., 2017; Lode et al., 2018), etc., while the Sr-containing salts used in the cement liquid mainly include Sr(NO_3_)_2_ (Lerous and Lacout, 2001) and SrCl_2_ (Tadier et al., 2012). Obviously, these abundant Sr-containing salts formed the various Sr-CPC systems without introducing any toxic substance *via* extensive ionic reaction routes. Comparing the main existing Sr-CPC systems listed in Table 1, there are many cement powder components consisting of triphase or multiphase powder system, such as β-TCP/Ca(H_2_PO_4_)_2_/SrCl_2_/Na_4_P_2_O_7_ system (Alkhraisat et al., 2008a), TCP/DCPA/CC/SC/HAP system (Schumacher et al., 2013), MCPM/Na_2_H_2_P_2_O_7_/β-TCP/SrF_2_(/SrI_2_/SrBr_2_/SrCl_2_⋅6H_2_O) system (López et al., 2014), TCP/DSPA/SC/HAP system (Baier et al., 2013), α-TCP/DCPA/SC/HAP/K_2_HPO_4_ system (Lode et al., 2018), and so on. Obviously, the triphase or multiphase cement system has over complicated preparation process. Their hydration processes are also difficult to control compared with that of single-phase or binary cement powder. In some other cement systems, the cement powder composition is simple, but they have many great shortcomings, such as complicated liquid formulations (Lerous and Lacout, 2001; Pina et al., 2010; Jayasree et al., 2017), high dose of harmful impurity introduced like Cl^–^ (Tadier et al., 2012) or NO_3_^–^ (Lerous and Lacout, 2001), longer setting time (Yu et al., 2009; Singh et al., 2016; Taha et al., 2017), and lower compressive strengths (Saint-Jean et al., 2005; D’Onofrio et al., 2016; Singh et al., 2016). In addition, as a special case, the binary Sr-β-TCP/MCPM system (Alkhraisat et al., 2008b) has been studied and shows a good releasing effect of Sr^2+^ ion, but the data about its mechanical properties is not available yet.

**TABLE 1:** Several Sr-CPC bone cements in the present available literatures.

Name	Composition	Hydration products	Major merits and demerits	Literatures
Sr-HAP bone cement	Powder phases: TTCP, DCPA, DSPA Liquid phases: PA	Ca_10__–_*_*m*_*_–_*_*x*_*Sr*_*x*_*__*m*_(HPO_4_)*_*y*_* (PO_4_)_6__–_*_*y*_*(OH)_2__–__2_*_*m*_*__2_*_*m*_* (0 < *x* < 1), nSr-HAP	Merits: higher compressive strength, 38.2∼66.5 MPa; *t*_I_:*t*_I_ = 4∼11 min; *t*_F_:*t*_F_ = 10∼17 min (PA > 0.5 mol L^–1^); no impurity in final product. Demerits: the data obtained *in vivo* is not enough.	Guo et al., 2005a
Injectable bioactive bone cement	Powder phases: Sr-HAP, reinforcing silica Liquid phases: D-GMA resin	Sr-HAP, D-GMA resin	Merits: excellent injectability and radiopacity. Demerits: lower compressive strength, 7.15 MPa; lower degradation rate for the resin *in vivo*.	Li et al., 2000
Calcium strontium HAP cements	Powder phases: TTCP, α-TCP Liquid phases: Strontium nitrate, orthophosphoric acid	Sr-HAP	Merits: easily prepared. Demerits: high dose of impurity ions (NO_3_^–^) contained in the final hardened body.	Lerous and Lacout, 2001
Sr-containing brushite cement	Powder phases: β-TCP, Ca(H_2_PO_4_)_2_, SrCl_2_, Na_4_P_2_O_7_ Liquid phases: 2M PA solution	Sr-DCPD, unreacted β-TCP	Merits: excellent cohesion and a diametric tensile strength of 5 MPa. Demerits: some Cl^–^ ions remained in the final hardened body and more than 3 powder phases.	Alkhraisat et al., 2008a
Sr modified biocements	Powder phases: Sr-β-TCP, MCPM Liquid phases: Water	Ca_(1__–__0.25_*_*x*_*_)_Sr_0.25_*_*x*_* HPO_4_⋅(*n*+1)H_2_O	Merits: good releasing effect of Sr^2+^ ions. Demerits: the data about its mechanical properties is not available.	Alkhraisat et al., 2008b
Sr-containing CPC	Powder phases: Sr-ACP, DCPA Liquid phases: deionized water	Sr-HAP, unreacted DCPA	Merits: higher compressive strength, 37∼74.9 MPa; porosity, 55.7∼58.2%. Demerits: the setting time is out of clinical requirement, *t*_I_ = 21∼28 min; *t*_F_ = 37∼52 min.	Yu et al., 2009
Newly developed Sr-substituted α-TCP bone cements	Powder phases: Sr-α-TCP Liquid phases: 10 wt.% poly(ethylene glycol), 20 wt.% citric acid solution; or 0.5 wt.% hydroxyl propyl methylcellulose, 10 wt.% poly(ethylene glycol), 20 wt.% citric acid solution	DCPD, unreacted Sr-α-TCP	Merits: expectable degradability. Demerits: lower compressive strength, 13.7 MPa; complex liquid phase composite.	Pina et al., 2010
Ca–Sr-mixed phosphate cement	Powder phases: DCPD, CaO, SC Liquid phases: ammonium phosphate buffer	Sr_1.35_Ca_7.65_(HPO_4_)*_*z*_* (PO_4_)_6__–_*_*z*_* (CO_3_)*_*z*_*_/2_	Merits: enhanced degradation rate of HAP. Demerits: lower compressive strength, 15.5∼20.1 MPa.	Romieu et al., 2010
A easy-to-prepare Sr(II)-modified CPC	Powder phases:α-TCP, DCPA, CC, SC, HAP Liquid phases: 4 wt.% Na_2_HPO_4_ aqueous solution	HAP, CC, SC, α-TCP, monetite	Merits: higher compressive strength, ∼57.7 MPa. Demerits: complex (5) phases in cement powder; longer setting time, *t*_I_ = 7.5∼15 or >15 min; *t*_F_ = 25∼34 min.	Schumacher et al., 2013
Sr-incorporated CPC	Powder phases: TTCP, DCPA, DSPA Liquid phases: a combination of citric acid and 12 wt.% polyvinylpyrrolidone K-30	Apatite	Merits: *t*_I_ = 6∼10 min, *t*_F_ = 13∼20 min. Demerits: lower compressive strength, 4∼14 MPa.	Kuang et al., 2012
Radiopaque brushite cements	Powder phases: MCPM, Na_2_H_2_P_2_O_7_, β-TCP, SrF_2_/SrI_2_/SrBr_2_/SrCl_2_⋅6H_2_O Liquid phases: distilled water	DCPD, β-Ca_2_P_2_O_7_, β-TCP, Monetite, unreacted SrF_2_	Merits: increased solubility; higher radiopacity. Demerits: lower wet compressive strength, <8 MPa; lower diametral tensile strength, <4 MPa. Complex phases in cement powder.	López et al., 2014
CPC	Powder phases: Sr-TTCP, DCPA Liquid phases: phosphate buffer solution, trisodium citrate	HAP, remaining TTCP	Merits: higher compressive strength, 38.66∼60.20 MPa; faster degradation rate; suitable setting time, 10∼17 min. Demerits: the data about its biocompatibility is not available.	Tao et al., 2013
Sr-enriched gelatin-CPC	Powder phases: gelatin-α-TCP, DCPD, SrCl_2_⋅6H_2_O Liquid phases: distilled water	α-TCP, calcium-deficient HAP	Merits: *t*_I_ = 4∼10 min, *t*_F_ = 8∼20 min (Sr% ≤2.0). Demerits: lower wet compressive strength, <13 MPa.	Panzavolta et al., 2008
Sr-containing CPC	Powder phases: TCP, DSPA, SC, HAP Liquid phases: an aqueous solution of 3 M K_2_HPO_4_ and 1.5 M KH_2_PO_4_	HAP	Merits: compressive strength, 34 MPa. Demerits: the data about its setting time is not available.	Baier et al., 2013
Sr-substituted α-TCP cements	Powder phases: Sr-α-TCP Liquid phases: 2.5wt.% Na_2_HPO_4_ accelerating solution	Sr-HAP, unreacted β-TCP.	Merits: good *in vitro* bioactivity. Demerits: lower compressive strength, <20 MPa.	Saint-Jean et al., 2005
Sr-loaded mineral bone cements	Powder phases: DCPD, CC, SC Liquid phases: deionized water or Powder phases: DCPD, CC Liquid phases: SrCl_2_⋅6H_2_O solution	CC, carbonated apatite, SC or CC, S-HAP	Merits: enhancing cell proliferation. Demerits: lower porosity.	Tadier et al., 2012
Sr-doped α-TCP bone cement	Powder phases: Sr-Ca_8_H_2_(PO_4_)_6_⋅5H_2_O, α-TCP Liquid phases: 100 mM citric acid solution	HAP	Merits: enhancing degradation and Sr ion release. Demerits: the data about its biocompatibility is not available.	Shi et al., 2017
Injectable CPC	Powder phases: ACP, DCPD, SC Liquid phases: deionized water	HAP, SC	Merits: increased injectability and compressive strength, 39.6 MPa. Demerits: the setting time is out of clinical requirement	Wang et al., 2007
CPC containing strontium ranelate	Powder phases: partially crystalline calcium phosphate, DCPA, strontium ranelate Liquid phases: deionized water	HAP, DCPA	Merits: good radiopacity and osteogenesis Demerits: the setting time is out of clinical requirement; lower compressive strength, <24 MPa.	Wu et al., 2019
Sr-modified premixed CPC	Powder phases: α-TCP, DCPA, SC, HAP, K_2_HPO_4_ Liquid phases: liquid consisted of Miglyol 812 with 14.7 wt.% Cremophor ELP and 4.9 wt.% Amphisol A	α-TCP, monetite, HAP, SC	Merits: enhancing mechanical properties; Better radiographic contrast. Demerits: complex powder and liquid phases composite.	Lode et al., 2018
A Sr-containing bioactive bone cement	Powder phases: Sr-HAP, fumed silica, benzoyl peroxide Liquid phases: a resin blend (Bisphenol A diglycidylether methacrylate, triethylene glycol dimethacrylate, poly(ethylene glycol) methacrylate, and *N*,*N*-dimethyl-p-toluidine)	Sr-HAP, resin	Merits: setting time, 15∼18 min; compressive strength, 40.9 MPa; bending strength, 31.3 MPa; Bending modulus, 1,408 MPa. Demerits: complex liquid phase composite.	Cheung et al., 2005
Sr-doped CPC	Powder phases: TTCP, DCPA, SC Liquid phases: ultrapure water	HAP, TTCP, SC	Merits: promoting osteogenic activity. Demerits: the data about its physicochemical properties and *in vivo* biocompatibility is not enough.	Huang et al., 2017
Sr-doped injectable bone cement	Powder phases: Sr-β-TCP, MCPM Liquid phases: water	DCPD, monetite, unreacted β-TCP	Merits: improved injectability; enhanced compressive strength. Demerits: *t*_I_ = 8∼16 min, *t*_F_ = 14∼25 min.	Taha et al., 2017
A novel injectable collagen-Sr-containing CPC	Powder phases: partially crystalline calcium phosphate, DCPA, modified starch Liquid phases: deionized water with type I collagen	HAP	Merits: improved antiwashout property and injectability; higher compressive strength, 21∼48 MPa. Demerits: *t*_I_ > 10 min, *t*_F_ > 15 min.	Zhou et al., 2015
Novel injectable Sr-hardystonite phosphate cement	Powder phases: Sr-doped hardystonite, NaH_2_PO_4_, Na_2_B_4_O_7_⋅10H_2_O Liquid phases: deionized water	Sr-doped hardystonite, willemite, silica	Merits: good injectability and handling properties. Demerits: *t*_I_ = 13.7 min, *t*_F_ = 21.3 min; lower compressive strength, ≤16 MPa; flexural strength, ≤3 MPa.	No et al., 2019
Sr releasing HAP forming cements	Powder phases: Sr-DCPD, TTCP Liquid phases: DI water or a 1.25% Na_2_HPO_4_ solution	Sr-HAP, TTCP	Merits: *t*_I_ = 4.0∼6.9 min, *t*_F_ = 6.3∼12 min (Na_2_HPO_4_ solution). Demerits: *t*_I_ = 18.5∼33.8 min, *t*_F_ = 26.2∼>60 min (DI water); Lower compressive strength <10.94 MPa; Decreased porosity.	Singh et al., 2016
Novel Sr containing bioactive glass based CPC	Powder phases: glass (SiO_2_-P_2_O_5_-CaO-SrO-Na_2_O), Ca(H_2_PO_4_)_2_ Liquid phases: 2.5% Na_2_HPO_4_ solution	Sr-HAP	Merits: increasing radiopacity. Demerits: lower compressive strength, <12.5 MPa; longer final setting time.	D’Onofrio et al., 2016
Sr and hydroxyl ion co-releasing radiopaque HAP cement	Powder phases: Sr-TTCP Liquid phases: 1 M Na_2_HPO_4_ and 10 wt.% citric acid	HAP, unreacted TTCP	Merits: *t*_I_ = 2∼5.5 min, *t*_F_ = 5∼10 min; compressive strength, 18.3∼32.4 MPa. Demerits: the data about its *in vivo* efficacy is not available.	Jayasree et al., 2017
Sr-incorporated biphasic CPC	Powder phases: Sr-β-TCP, TTCP Liquid phases: PA	Sr-β-TCP, Sr-HAP	Merits: suitable operational properties; excellent washout resistance; *t*_I_ = 4∼8 min, *t*_F_ = 10∼15 min; Compressive strength, 46.6 MPa. Demerits: the data about its *in vivo* efficacy is not available.	Zhu et al., 2017

Tricalcium phosphate, as a biomedical ceramic or powder, has excellent biocompatibility, osteoconductivity, degradation property, and bone inductivity, which has been widely used in clinics. Based on the formation temperature, TCP is divided into a low-temperature phase (β-TCP) and a high-temperature phase (α-TCP). Beyond 1,120°C, β-TCP could transform to α-TCP. As a high-temperature phase, α-TCP possesses high hydration reaction activity, high-temperature stability (stably in range of 1,120∼1,470°C) and can be hydrated into apatite easily under alkaline condition (Dorozhkin and Epple, 2002). As a comparison, α-TCP degrades much faster than HAP, β-TCP, and even mixture of HAP/β-TCP (Carrodeguas and De, 2011). So it is usually used as a component to create new biocomposite or bone cement powder system. Although α-TCP possesses many excellent biological properties, it is still far from the actual demand of clinic in a single-phase form due to its too-fast degradation (Dai et al., 2011).

In order to improve service capacities and enlarge clinical application scopes of α-TCP, recent studies mainly focus on improving the physicochemical properties and biocompatibility by doping ions including Zn^2+^ (Li et al., 2009), Mg^2+^ (Famery et al., 1994), Fe^2+^ (Fernández et al., 2005), SiO_4_^4–^ (Martínez et al., 2010), Ba^2+^ (Yashima and Kawaike, 2007), etc., into α-TCP. Especially, to dope Sr element into α-TCP not only provides a strategy to modify its physicochemical properties but also yields a new Sr-containing salt (Sr-α-TCP). It was also proved that the addition of Sr-α-TCP into Sr-β-TCP/TTCP system significantly accelerated the hydration and also increased the compressive strength of the hardened body (Zhu et al., 2017). At present, Sr-α-TCP was mainly synthesized by using two-step methods (Saint-Jean et al., 2005; Pina et al., 2010). For example, Saint-Jean et al. (2005) firstly prepared Ca_2_P_2_O_7_ by firing DCPA at 1,100°C for 16 h and then gained Sr-α-TCP by sintering mixture powders of CC, SC, and Ca_2_P_2_O_7_ at 1,500°C. Also, Pina et al. (2010) firstly prepared Sr-β-TCP by aqueous precipitation mixing with Ca(NO_3_)_2_⋅4H_2_O, Sr(NO_3_)_2_, and (NH_4_)_2_HPO_4_ and then sintered the Sr-β-TCP powder at 1,500°C for 2 h to get Sr-α-TCP. Both methods are over complicated, and the sintering temperature is as higher as 1,500°C. Thus, an easier method to prepare Sr-α-TCP is introduced in this study.

In this study, we synthesized an important Sr-containing salt, Sr-α-TCP, with a simpler one-step method and relatively lower synthesis temperature. Moreover, we used the as-prepared Sr-α-TCP to develop a new Sr-CPC system based on the above Sr-CPC designing strategies. A binary TTCP/Sr-α-TCP mixture was used as the cement powder and a simple diluted PA solution was used as the cement liquid. So far, this TTCP/Sr-α-TCP system has not been proposed and thus its properties have not yet been reported. Here, the *in vitro* physicochemical properties, including final hydration product composition, setting time, compressive strength, microstructure, hydration crystal morphology, entire phase-evolution process, and initial cytocompatibility tests were investigated.

## Materials and Methods

### Preparation of the Sr-CPC Powder Mixtures

As mentioned above, the Sr-CPC powders were a biphasic mixture composed of Sr-α-TCP and TTCP. The Sr-α-TCP was prepared by directly sintering the starting mixture of DCPA, CC, and SC (see Table 2), followed by a sintering step at 1,400°C for 10 h in a furnace, where the heating units were silicomolybdic rods. The starting mixture for preparing Sr-free α-TCP was only composed by DCPA and CC with a molar ratio of 2:1. The interrelated chemical equation for preparing Sr-α-TCP can be described as follows: 2CaHPO_4_ + (1–*x*) CaCO_3_ + *x*SrCO_3_ = α-Sr*_*x*_*Ca_1__–_*_*x*_*(PO_4_)_2_ + H_2_O + CO_2_. After that, the heated mixture was quenched to room temperature in a desiccator. The as-prepared product was then determined by XRD analysis. The obtained Sr-α-TCP or Sr-free α-TCP products were crushed using mortar and pestle and then wetly ball-milled for 12 h with liquid medium of absolute ethanol. The milling conditions were described as follows: the materials of ball and jar (200 ml) were aluminum oxide and nylon, respectively; 100 smaller balls in Φ5 mm and 15 larger balls in Φ10 mm were used for each jar. The total mass of the raw powders for each jar was 50 g, and the average particle size is around 30∼50 μm. The rotation speed was set at 400 r min^–1^. The device used here was planet ball-milling machine produced by Nanjing Uni. Instrument Co., China. The mean size of the grounded particles was measured with the light scattering particle size analyzer (Winner 2000, China). The TTCP was prepared by heating the starting mixture of DCPA and CC in a Ca/P atom ratio of 2.0 by the experimental route similar to the Sr-α-TCP. However, the sintering temperature and the sintering time is changed to 1,500°C and 15 h (Guo et al., 2005b), respectively. After being quenched to the room temperature, the obtained TTCP products experienced a similar wetly ball-milling route mentioned above and the mean size of the grounded TTCP particles was 14.0 ± 4.5 μm. The final cement powder mixtures consisted of Sr-α-TCP and TTCP with a molar ratio of 2:1.

**TABLE 2:** Syntheses of various α-TCP or Sr-α-TCP.

Sample name	Composition of starting powder	Mean size of the grounded α-TCP particles
	SC:DCPA:CC	
0%Sr-α-TCP	0:2:1	7.9 ± 2.3 μm
8.3%Sr-α-TCP	0.25:2:0.75	8.3 ± 1.9 μm
16.7%Sr-α-TCP	0.50:2:0.50	9.5 ± 2.6 μm

### Preparation of the Sr-CPC Samples

The Sr-CPC pastes were prepared by mixing biphasic Sr-CPC powders and PA solution (0.75 mol L^–1^) with a suitable P/L (1.8) using a spatula for 30 s. After mixing, the obtained pastes were placed into a stainless steel mold (6 × 12 mm in diameter and height) with a pressure of 0.70 MPa. The compositions of the Sr-CPC pastes are described in Table 3. After shaping, the cement columns were stored in an incubator at 37°C with 100% relative humidity for 15 min and were then immersed into SBF (Kokubo, 1991; Kokubo and Takadama, 2006) for 72 h. Finally, the immersed columns were taken out and dried 30 min in an oven at 37°C for the following measurements. To observe their microstructures using FESEM (S-2700, Japan), the dried columns were directly crashed into fragments, and some of the larger ones were selected. The residual fragments were further grounded into fine powder for measuring the composition of the hydration products with XRD analysis apparatus. Besides, pH values of the SBF before and after different samples immersion were measured by pH meter (PHSJ-4A, China). Totally, three kinds of Sr-CPC samples with a different Sr/(Sr+Ca) molar ratio, defined by 0Sr-CPC (0%), 5Sr-CPC (5%), and 10Sr-CPC (10%), respectively, were prepared in the present study (the details were shown in Table 3).

**TABLE 3:** Compositions of various cements and pH values for the SBF after different-sample immersion.

Sample name	Cement powder		Sr/(Sr+Ca)	pH values for the SBF after different samples immersion
	(molar ratio: A:B = 2:1)			
	A	B		
0Sr-CPC	0%Sr-α-TCP	TTCP	0%	7.430
5Sr-CPC	8.3%Sr-α-TCP	TTCP	5%	7.433
10Sr-CPC	16.7%Sr-α-TCP	TTCP	10%	7.537

### Compressive Strength Measurement

The compressive strength values of the hardened samples were measured by using a universal testing machine (WDW-1000, China). The loading rate was controlled at 1 mm min^–1^ for each cement sample. Five parallel tests at least were used to obtain the mean compressive strength value and the responding standard deviation for each sample. The hydration parameters selected for investigating their effects on compressive strength of certain Sr-CPC cement were shown in Table 4. Before measuring compressive strength, the samples were fixed by a special clamp and then both ends of samples were polished using a fine abrasive paper. The obtained dimension of the polished samples is approximately 6 mm for diameter and 11.5 mm for height.

**TABLE 4:** Hydration parameters of various Sr-CPC cements and their effects on the setting time.

Sample name	L.C. (mol L^–1^)	P/L ratio	Sr/(Sr+Ca) (×100%)	Setting time (min)	
				*t*_I_	*t*_F_
0Sr-CPC-a	0.75	1.8	0%	1.5 ± 0.2	7.0 ± 0.3
5Sr-CPC-b	0.75	1.8	5%	1.5 ± 0.1	7.5 ± 0.5
10Sr-CPC-c	0.75	1.8	10%	1.5 ± 0.1	8.0 ± 0.1
10Sr-CPC-d	0.5	2.0	10%	2.0 ± 0.2	16.0 ± 0.1
10Sr-CPC-e	1.0	1.6	10%	1.0 ± 0.1	4.0 ± 0.4

### Setting Time Test

The setting times, including the initial time and the final time, of various cements were determined using Gil-more needles according to the C266-99 ASTM standard (Schumacher et al., 2013). In this method, the cement samples were stored at 37°C with a relative humidity of 100%. The processing details of the sample for testing the setting times were described as follows: the cement pastes were prepared by mixing the cement powder with the PA solution at a certain weight ratio using a spatula. After 30 s, the cement pastes were put into a quartz glass mold (6 × 12 mm) with a pressure of 0.70 MPa and then immediately stored in the incubator at 37°C (100% relative humidity). The cement pastes were taken out to check their setting status every 1 min. Generally, six parallel tests at least were used to obtain the mean value and the responding standard deviation for each sample. The apparatus used here was the Cement Standard Consistency and Setting Time Device (Lu-Xun Co., China). The hydration parameters selected for investigating their effects on setting time of certain Sr-CPC cement were the same to those shown in Table 4.

### Real-Time Evolutions in Phase Composition and Microstructures of the Sr-CPC Cement

To investigate the hydration process of this novel bioactive bone cement, 10Sr-CPC-c pastes after being immersed in SBF for 30 min, 1, 2, 6, 10, 15, 24, and 72 h, respectively, were terminated using acetone solution refrigerated at −16°C, and then the samples were taken into an oven at 50°C for drying. The phase composition and microstructures of the Sr-CPC samples hydrated at various stages were measured and observed by XRD analysis apparatus and FESEM, respectively. Besides, the compressive strength at various hydration stages were measured by the similar method described in “Materials and Method.”

### Characterization Methods

Crystalline phases of the powders were determined by XRD with Cu-K radiation and Ni filter. The electrical voltage and current were 35 kV and 30 mA, respectively. The fractured surfaces of the cement samples were examined with SEM (JSM6460, Japan) and the microscopic morphologies of the hydration crystals were observed with FESEM. Chemical element on the fractured surface of cement samples were detected with EDS (JSM6460, Japan) which provided by SEM.

### Testing of Cell Behaviors

#### Cell Culture

Osteoblast-like MC3T3-E1 subclone 4 were cultured in a-MEM (Hyclone, United States) containing 100 units ml^–1^ penicillin and 100 μg ml^–1^ streptomycin and supplemented with 10 vol.% fetal bovine serum (Hyclone, United States) at 37°C with 5% CO_2_. Osteogenic medium was prepared by adding 10 mM β-glycerophosphate (Sigma-Aldrich, United Kingdom) and 50 mg ml^–1^ ascorbic acid (Sigma-Aldrich, United Kingdom) into culturing medium for the alkaline phosphatase test.

#### Cytotoxicity

Cytotoxicity tests were performed by adding different dilutions of sample extract to a MC3T3-E1 cell culturing on a 96-well plate. Samples were extracted in serum-free a-MEM according to ISO 10993. The ratio of sample weight to cell culture medium volume was 0.2 g ml^–1^. The resulting extract was serially diluted with cell culture medium and supplemented with 10 vol.% fetal bovine serum. Cells were seeded into 96-well culture plates at a cell density of 5,000 cells per well. After overnight incubation, the cell culture medium was subsequently replaced with sample extract or cell growth medium (negative control) (*n* = 5), and the cells were incubated for an additional 1 or 3 days. Then cell viability was assayed by MTT assays (Chen et al., 2013). After 1 or 3 days, the culture medium was discarded and 100 μl of 5 mg ml^–1^ MTT in phosphate buffer saline was added to each well. The cells were incubated in the dark for 3 h at 37°C and 10% CO_2_. Then, the MTT solution was discarded and the insoluble formazan was dissolved with dimethyl sulfoxide for 20 min at room temperature. The absorbance was measured against blank at a wavelength of 570 nm by a microplate reader (Spectra Max 250, MWG Biotech). Results were reported as means and standard deviations of the measured absorbance normalized to the absorbance of negative control for the MTT assay.

#### Cell Proliferation

Disk samples with dimensions of 10 mm in diameter and 1 mm in thickness were prepared, briefly submersed in 70% ethyl alcohol and allowed to dry under sterile conditions for all experiments. TCPS plates were used as positive control. The pre-incubated cell line was placed on samples (*n* = 3) for each group of bone cement at densities of 2 × 10^4^ cells cm^–2^ for the cell proliferation tests. Methoxyphenyl tetrazolium salt assay was used to evaluate cell numbers after cultured in a humidified atmosphere of 5% CO_2_ at 37°C for 1, 4, and 7 days in growth medium.

## Results

### Synthesis of Sr-α-TCP

**FIGURE 1:**
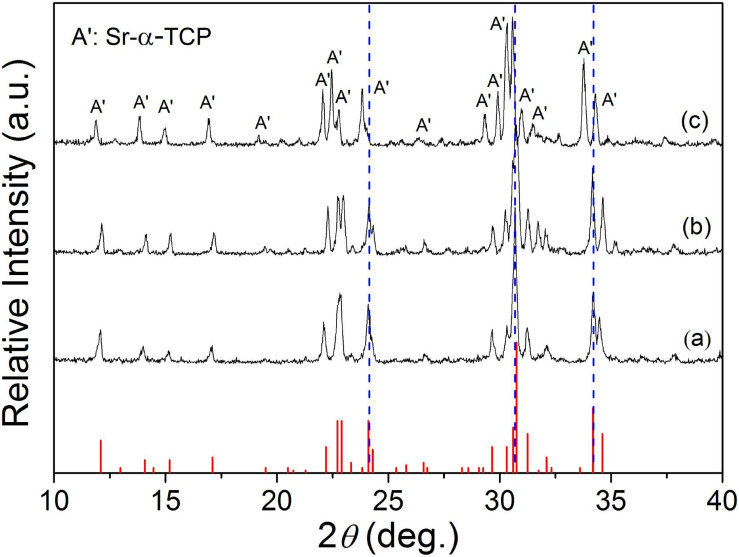
The XRD patterns of α-TCP containing different Sr contents: (a) α-TCP; (b) 8.3%Sr-α-TCP; and (c) 16.7%Sr-α-TCP.

**FIGURE 2:**
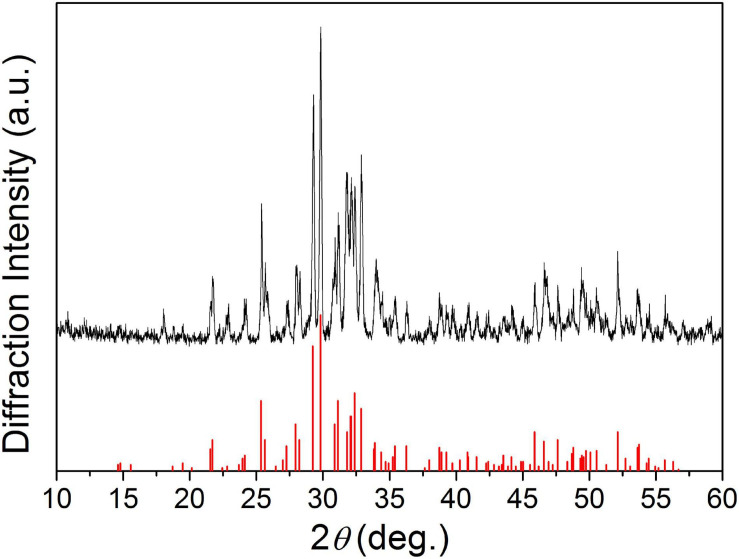
The XRD pattern of Ca_4_(PO_4_)_2_O.

Figure 1 shows the XRD patterns of the final sintered products (α-Sr*_*x*_*Ca_3__–_*_*x*_*(PO_4_)_2_, *x* = 0, 0.25, and 0.50) from the starting cement powder with different compositions shown in Table 2. The diffraction peaks and angles of the Sr-free product [Figure 1(a)] were well consistent to those of α-TCP according to JCPDS no. 09-0348 and no other impurity phase was detected. Obviously, the Sr-free product is the pure single phase α-TCP. The diffraction peaks of two obtained Sr-contained products [Figures 1(b,c)] were also similar to those of pure α-TCP without any detected impurity phase. Only a slight difference was observed on their diffraction angles. The diffraction angles of Sr-contained products slightly shifted toward left in comparison with the Sr-free product. The increased Sr ratio from 8.3 to 16.7% led to a further shift. Based on the above analyses and the designed composition of the starting materials shown in Table 2, it could be referred that the products corresponding to Figures 1(b,c) are α-Sr_0.25_Ca_2.75_(PO_4_)_2_ (8.3%Sr-α-TCP), and α-Sr_0.50_Ca_2.50_(PO_4_)_2_ (16.7%Sr-α-TCP), respectively. The mean sizes of the grounded α-TCP, 8.3%Sr-α-TCP and 16.7%Sr-α-TCP particles are also shown in Table 2. In addition, the XRD pattern of as-prepared products shown in Figure 2 was proved to be pure TTCP phase (Guo et al., 2005b) according to JCPDS no. 25-1137.

### Hydration Products of Various Cement Samples

**FIGURE 3:**
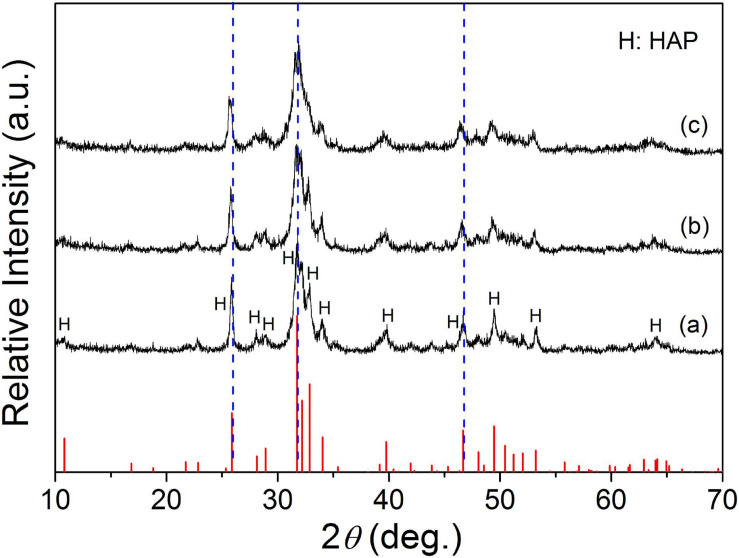
XRD patterns of various Sr-CPC samples after immersed in SBF at 37°C for 72 h: (a) 0Sr-CPC-a; (b) 5Sr-CPC-b; and (c) 10Sr-CPC-c.

Figure 3 shows the diffraction patterns of hydration products corresponding to the Sr-CPC samples with different Sr contents. It was obvious that the diffraction patterns of all the hydration products including 0Sr-CPC, 5Sr-CPC, and 10Sr-CPC were similar with that of HAP (JCPDS no. 09-0432). The only difference was that their diffraction peaks gradually shifted toward left when the Sr content in cement varied from 0 to 10%. It implied the hydration products of Sr-CPC samples with different Sr contents were Sr^2+^-substituted HAP. The shift of the diffraction peaks were caused by the variation of crystal interplanar spacing induced by the incorporation of Sr ions (Sun et al., 2012, 2014). The pH value of the prepared SBF is 7.400 and pH values for the SBF after 0Sr-CPC, 5Sr-CPC, and 10Sr-CPC immersion were 7.430, 7.433, and 7.537, respectively. It indicated that the immersion of different Sr-CPC samples had no obvious effect on the pH values of SBF.

**FIGURE 4:**
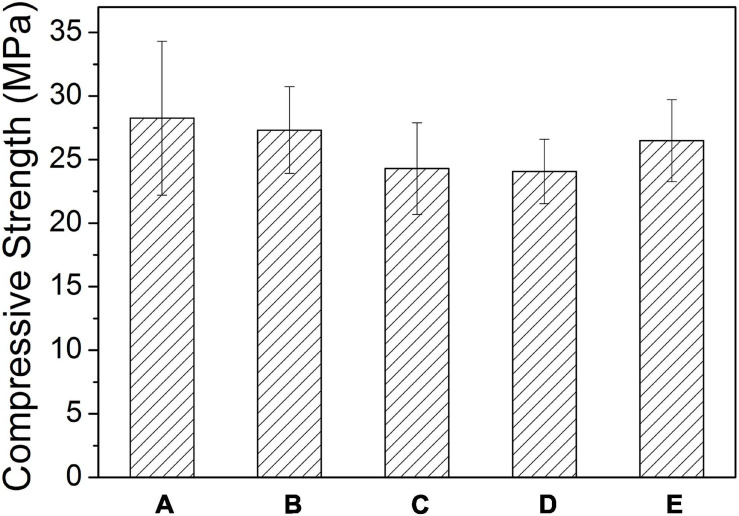
Compressive strength values of Sr-CPC cements with different hydration parameters listed in Table 4 after immersed in SBF at 37°C for 72 h: (a) 0Sr-CPC-a; (b) 5Sr-CPC-b; (c) 10Sr-CPC-c; (d) 10Sr-CPC-d; and (e) 10Sr-CPC-e.

Figure 4 is the tested compressive strength values of the Sr-CPC cements with different hydration parameters listed in Table 4. Before testing, all the samples experienced a 72-h immersion process in SBF at 37°C. Comparing the data of Figures 4(a–c), the compressive strength values of the Sr-CPC cements slightly decreased from 28.26 ± 6.04 to 24.30 ± 3.61 MPa with the Sr content increased from 0 to 10% (*P* < 0.05). In addition, comparing the data of Figures 4(c–e), the compressive strength values of 10Sr-CPC-c were extremely close to that of 10Sr-CPC-d but slightly lower than that of 10Sr-CPC-e (*P* < 0.05). The above results indicate the effect of the hydration parameters, including Sr content, L.C., and P/L ratio, on the compressive strength values of the biphasic Sr-CPC is not remarkable. In addition, the setting time including *t*_I_ and *t*_F_, of various cements determined using Gil-more needles is shown in Table 4. For various hydration parameters, *t*_I_ and *t*_F_ of the biphasic Sr-CPC ranged from 1.0∼4.0 to 7.0∼16.0 min, respectively. In comparison with the triphasic Sr-CPC with a TTCP/DCPA/DSPA cement powder system reported previously (Guo et al., 2005a), this biphasic Sr-CPC cement possessed a characteristic of fast-setting.

**FIGURE 5:**
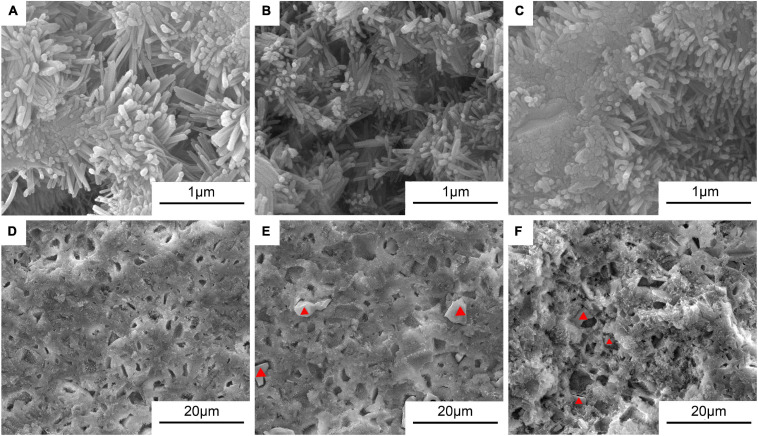
The SEM morphologies in the typical areas on the fractured surfaces of various Sr-CPC cements after immersed in SBF at 37°C for 72 h: **(A,D)** 0Sr-CPC-a; **(B,E)** 5Sr-CPC-b; and **(C,F)** 10Sr-CPC-c.

Figure 5 shows the typical morphologies on the fractured surfaces of the cement samples including 0Sr-CPC-a, 5Sr-CPC-b, and 10Sr-CPC-c. After a 72-h immersion in SBF, many pores with a similar size of 10∼30 μm appeared on fractured surface of each cement. At a higher magnification, it could be clearly observed that each fractured surface was composed of different twisting nanocrystals with a similar size (60 × 500 nm). Additionally, no other obvious difference except for some ceramic-like particles was found on the fractured surfaces of Sr-containing cement samples (5Sr-CPC-b and 10Sr-CPC-c). The corresponding data of EDS spectra of these ceramic-like particles are shown in Table 5. The result indicates that these ceramic-like particles consisted of Ca, Sr, P, and O and the molar ratio of (Sr+Ca)/P is close to 1.5. Thus, it is referred that the ceramic-like particles are the residual Sr-α-TCP after hydration.

**TABLE 5:** EDS data on the surface of the ceramic-like particles in the 10Sr-CPC-c cement after being immersed in SBF at 37°C for 72 h.

Element	Weight %	Atomic %
*O*_*K*_	41.14	63.46
*P*_*K*_	18.75	14.94
Ca_*K*_	30.83	18.99
Sr_*L*_	9.28	2.61
Total	100.00	100.00

### The Real-Time Hydration Process of Sr-CPC

**FIGURE 6:**
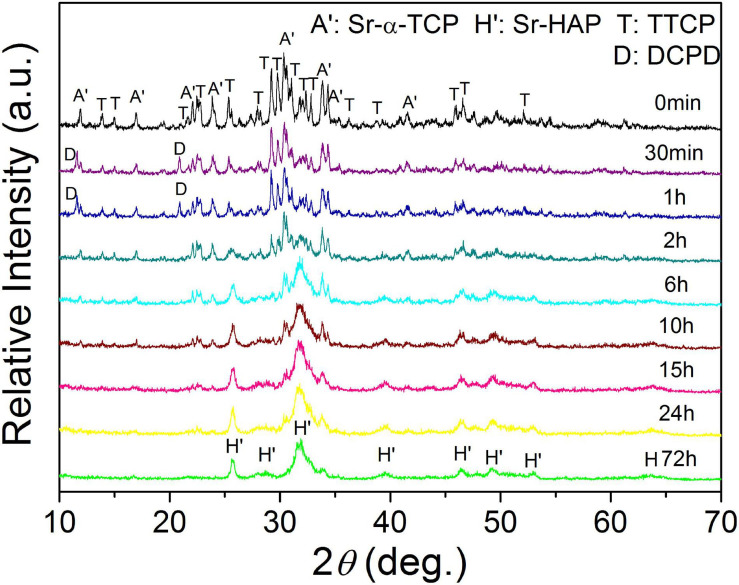
The real-time XRD patterns of the 10Sr-CPC-c samples after immersed in SBF.

The XRD patterns of 10Sr-CPC-c hydrated at different stages in SBF are shown in Figure 6. During the initial hydration period from the starting time to 2 h, no obvious new diffraction peak of hydration product was detected. Only a slight decrease in the intensities of the major diffraction peaks corresponding to TTCP and Sr-α-TCP was observed. However, all the peak intensities of TTCP and Sr-α-TCP conspicuously decreased after 2 h and the diffraction peaks of TTCP were difficult to be identified till 6 h. Simultaneously, the diffraction peaks of apatite, located at 2θ = 25.8° and 31.8° corresponding to crystal plane (002) and (211), could be clearly detected at 6 h and gradually turned stronger as the hydration proceeded. At 72 h, the main diffraction peaks of apatite near 2θ = 31.8° overlapped together like a wave hump, indicating a poor crystallinity for the hydration product. Interestingly, the diffraction peaks of Sr-α-TCP could be traced till 24 h and finally disappeared at 72 h.

**FIGURE 7:**
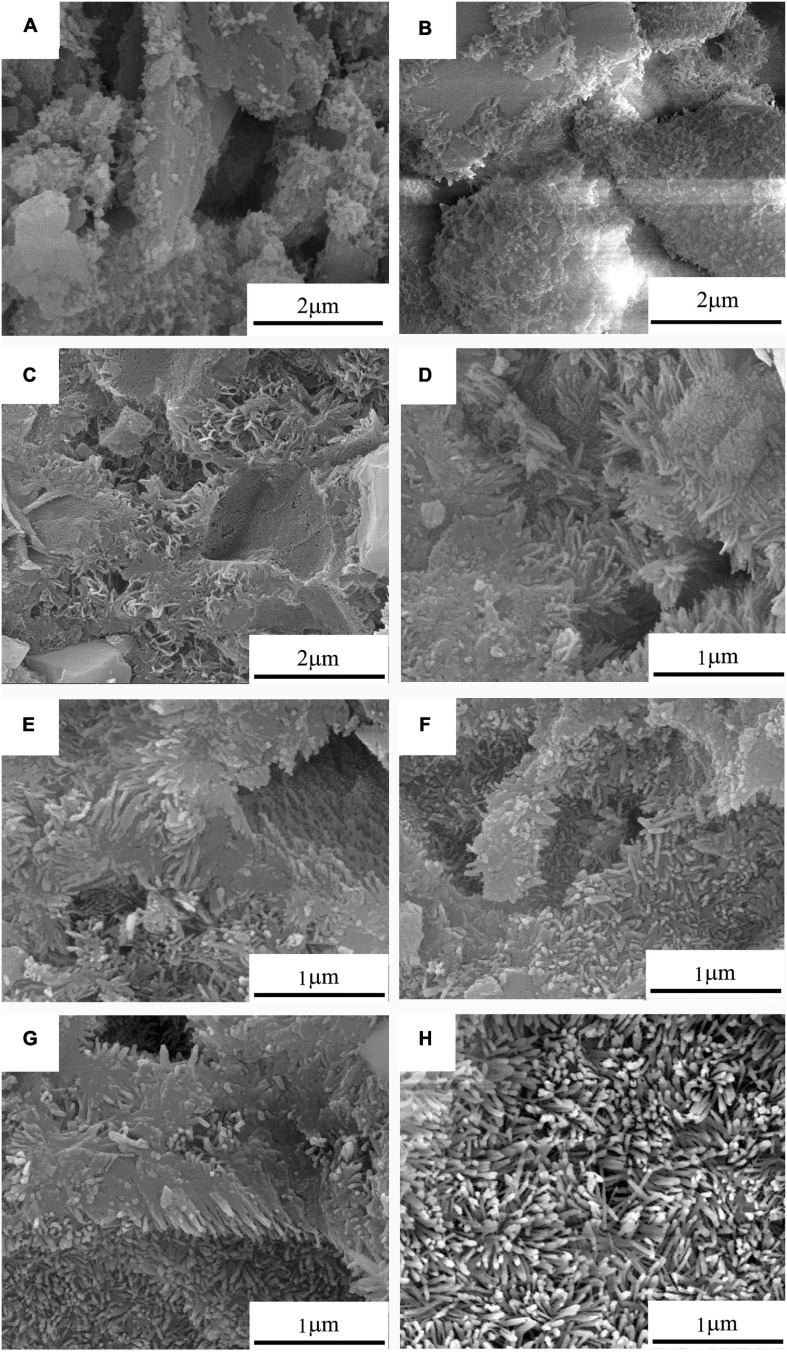
The real-time FESEM photographs of 10Sr-CPC-c samples after hydrating in SBF for different time: **(A)** 30 min; **(B)** 1 h; **(C)** 2 h; **(D)** 6 h; **(E)** 10 h; **(F)** 15 h; **(G)** 24 h; and **(H)** 72 h.

The corresponding FESEM photographs of 10Sr-CPC-c hydrated at different stages in SBF are shown in Figure 7. Before the hydration reaction started, no other new change was found on the cement powder mixture. At 30 min, many small projections in a nanoscale started to appear on the part of the smooth surfaces of the dispersed ceramic-like particles in a micron scale. It means a just beginning of the hydration reaction at this time point. However, at 2 h, these larger dispersed ceramic-like particles had already been wielded by a kind of sheet-like crystals in a nanoscale. As a matter of fact, after this time point, the larger ceramic-like particles with a dense structure had gradually been replaced by a kind of loose structure composed with such sheet-like crystals. At 6 h, the nanosheet-like crystals had gradually transferred into twisting nanorod-like crystals with a diameter of 20∼30 nm and length of 200∼300 nm (see Figure 7D). When the hydration reaction proceeded, the contour profile of the twisting nanorod-like crystals became clearer and slightly increased (size 30∼40 × 300∼500 nm, Figure 7H). After 24 h, the hydration products present with two typical parts: dense part and loose part. The dense part is a block-like structure composed of tightly packed nanorod-like crystals, while the loose part consisted of abundant twisting nanorod-like crystals with a certain volume of pores in a nanoscale.

**FIGURE 8:**
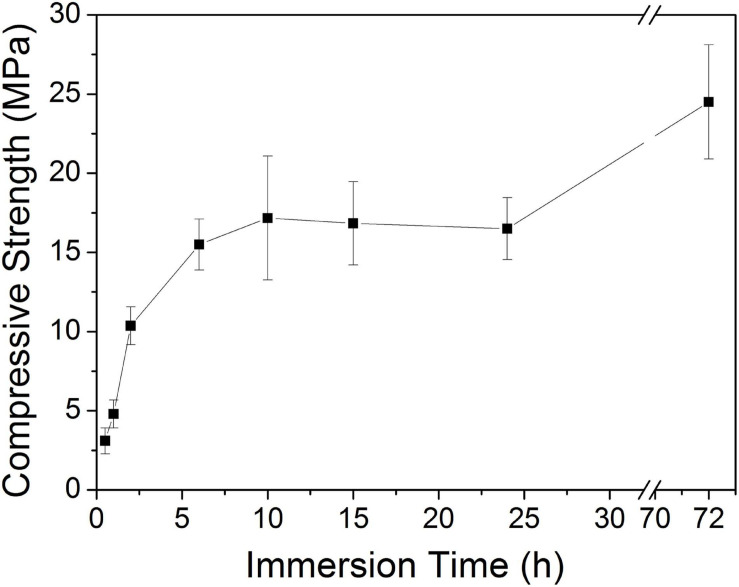
Compressive strength evolution curves of the 10Sr-CPC-c samples immerse in SBF for different hydration stages.

Figure 8 shows the tested compressive strength values of the 10Sr-CPC-c samples immersed in SBF for different hydration stages. The biphasic cement yielded a considerable compressive strength of 10.4 ± 1.12 MPa at 2 h. The compressive strength remained stable in the range of 15.5∼17.2 MPa as the hydration time varied from 6 to 24 h. Finally, this 10Sr-CPC-c achieved a maximal compressive strength value of 24.5 ± 3.61 MPa at 72 h.

### Evaluation of Cell Behaviors

**FIGURE 9:**
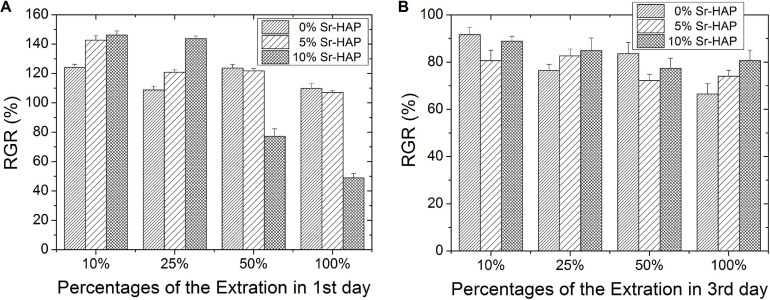
RGR of MC3T3-E1 cells cultured in different concentrations of extract for **(A)** 1 day and **(B)** 3 days.

Figure 9 displays the results of cytotoxicity test *in vitro*. Figures 9A,B represents the relative growth rate (RGR) of MC3T3-E1 cells cultured in different dilutions of sample extract for 1 and 3 days, respectively. The RGR at extract concentration of 10% was much higher than that at extract concentration of 100%, which was consistent with a previous report (Cahn, 1988). As shown in Figure 9A, the RGR of both 5%Sr-HAP and 10%Sr-HAP were higher than that of 0%Sr-HAP after cells were cultured in extract with concentration of 10 and 25% for 1 day, indicating that Sr doping would lead to a lower cytotoxicity, especially for the 10%Sr-HAP. However, 10%Sr-HAP possessed the highest cytotoxicity at extract concentration of 50 and 100%. As can be seen in Figure 9B, the RGR in all groups decreased after cells were cultured for 3 days, ranging from 70 to 90%. On the whole, with the increase of the concentration of extract, the cytotoxicity gradually increased in all group.

**FIGURE 10:**
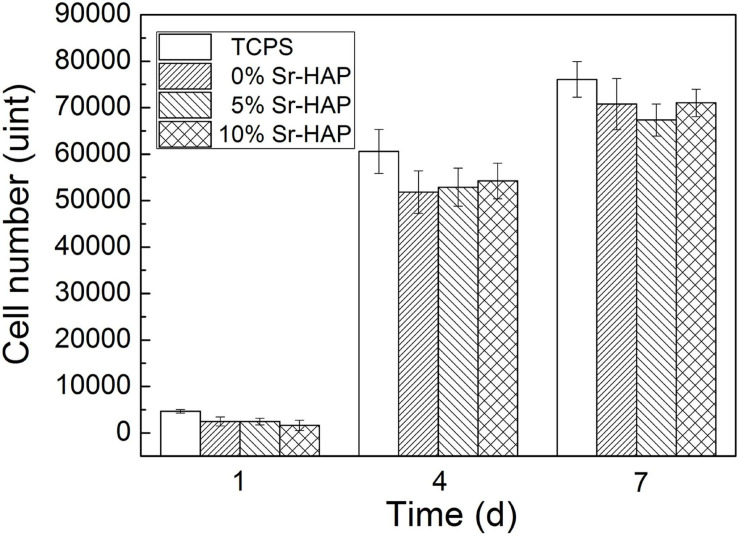
Cell number of MC3T3-E1 cells proliferated on the surface of the samples for 1, 4, and 7 days.

Figure 10 shows the proliferation of MC3T3-E1 cells cultured on the surface of the samples for 1, 4, and 7 days. TCPS was the standard control group. After being proliferated for 1, 4, and 7 days, there was no significant difference in the cell number in each groups. It was inferred that the addition of Sr had no effect on cell proliferation in the present doses. With the extension of time, the cell number increased continuously, indicating that the Sr-CPC had great cellular compatibility.

## Discussion

Since being invented some 30 years ago, CPC have attracted a lot of interests as bone replacement material. The CPC contains two important parts: the cement powder phase and the cement liquid phase. Any part of them does play an important role in their hydration reaction, physicochemical properties and cytocompatibility. As is well known, a complicated composition of the cement powder or cement liquid will cause difficulty in controlling the hydration reaction, which may result in higher cost of the product. In view of this, an important Sr-source salt, Sr-α-TCP, were firstly synthesized with a simplified one-step method and relatively lower synthesis temperature compared with the existing ways (Saint-Jean et al., 2005; Pina et al., 2010). Based on the above Sr-CPC design strategies, we developed a new Sr-CPC system with a binary TTCP/Sr-α-TCP mixture used as the cement powder and a simple diluted PA solution used as the cement liquid. Moreover, as an initial assess on this novel Sr-CPC system, its *in vitro* physicochemical properties and cytocompatibility were investigated in the present study.

It has been reported that doping xenogeneic ions into lattices will lead to lattice distortion and variation of the lattice constants due to the different radius between xenogeneic ions and substrate ions (Shi, 2003). In this study, Sr ions with a larger radius were doped into lattice of α-TCP to replace equivalent Ca ions, and lattice distortion or lattice expansion happened. According to the Bragg’s equation λ =  2*d*sinθ, the diffraction angle of Sr-α-TCP is smaller than that of α-TCP. In view of this, the left shifted of diffraction peaks (Figure 1) have testified that Sr ions had been successfully incorporated into the lattices of α-TCP by replacing equivalent Ca ions and not just adsorbed on its crystal surface or aggregated in the crystal boundary. Similarly, Figure 3 shows that with the increase of Sr content, all the diffraction peaks of the hydration products shifted toward left consistently, which also indicated that Sr ions were doped into the HAP lattice and the hydration products were Sr-HAP. Based on this, the strategy to design a Sr-containing salt and to gain a Sr-CPC is practical.

According to the results of Figures 4A–C, a negligible decrease of the mean compressive strength of Sr-CPC was observed with the increase of Sr content. In addition, the incorporation of the Sr-α-TCP into the cement powder significantly delayed its hydration reaction. This is attributed to its higher degree of supersaturation for yielding apatite crystals and lower transformation rate of Sr-HAP, when exposed to the Sr-containing hydration system compared with Sr-free HAP cement paste (Christoffersen et al., 1997; Guo et al., 2011). Raw cement powder constantly reacted to form HAP and hardened during the hydration process of Sr-CPC. Thus, the delay effect of Sr ions on the transformation rate of HAP will not largely affect the strength of hydration products. The results were also well consistent with those reported elsewhere (Kuang et al., 2012; Zhu et al., 2017; Wu et al., 2019) that the addition of Sr ions reduced the compression strength of Sr-CPC. Some ceramic-like particles were found in the hydration products of 5Sr-CPC (Figure 5E) and 10Sr-CPC (Figure 5F), but not found in 0Sr-CPC (Figure 5D), and the ceramic-like particles were confirmed to be the residual Sr-α-TCP (see Table 5). This provides another morphological proof that confirmed the delay effect of Sr ions on the hydration reaction of Sr-CPC. In addition, in the present study, SBF provides a physiological-like environment for the hydration of Sr-CPC as an immersion solution. During immersion, SBF provides sufficient H_2_O, Ca^2+^, and PO_4_^3–^ ions for the Sr-CPC sample and accelerates the further hydration of cement. Therefore, the SBF-immersion is in favor of the increase of CPC compression strength. The compressive strengths of the other Sr-α-TCP-based cement systems reported elsewhere were 13.7 MPa (Pina et al., 2010) and about 16 MPa (Saint-Jean et al., 2005), which were much lower than that of the TTCP/Sr-α-TCP cement system developed in this study (24∼27 MPa).

But there is an easily roused question why the residual Sr-α-TCP ceramic particles did not play an *in situ* reinforcing effect on the cement-hardened body? In the previous study (Guo et al., 2006), after immersed in SBF for 72 h, there was a certain amount of unreacted TTCP remained in the TTCP/DCPA bone cement system with physiological saline solution as cement liquid. This CPC achieved the highest compression strength of 103 MPa at 72 h in SBF attributed to the *in situ* reinforcing effect of the residual TTCP. As a kind of alkaline phosphate, TTCP hydrated partly in the physiological saline solution and the hydration product tightly covered the surface of the residual TTCP particles after immersing in SBF for 72 h. Hence, the bonding force between the hydration product (nanoscale HAP particles) and the residual TTCP particles was strong and the *in situ* reinforcing effect of the residual TTCP ceramics was very remarkable. However, in this study, at the immersed stage in SBF of 72 h, the dissolution rate of the residual Sr-α-TCP particles was much faster than the formation rate of hydration products due to the delay effect of Sr, which yielded gaps and pores between hydration products and Sr-α-TCP particles (see the red triangles shown in Figure 5). As a result, these gaps and pores weakened the bonding force between the hydration product and the residual particles and thus the increased porosity reduced the compression strength of the Sr-CPC system. In other words, it follows that not all the residual particles played an *in situ* reinforcing role on the hardened cement matrix. However, from a viewpoint of cytobiology, the increase of porosity is beneficial to the growth of new bone (Karageorgiou and Kaplan, 2005; Hannink and Arts, 2011).

It was also found that the diluted PA is another important parameter affecting the compression strength of the Sr-CPC. As shown in Figures 4C–E, with the increase of L.C., the compression strength of the Sr-CPC gradually increased. As a cement liquid, the diluted PA itself provides more PO_4_^3–^ and higher acidity than other cement liquid (Guo et al., 2011). Hence, with the increase of L.C., the alkaline TTCP hydrated fastly, and the transformation rate of Sr-HAP increased. Based on the above results, the compressive strength of this Sr-CPC could be improved by coordinating the Sr^2+^ content and the PA concentration in the cement system.

From the viewpoint of the rheological properties of the cement pastes, the physical nature of the Sr-CPC setting process can be expressed as follows (Shen et al., 1998): In the initial setting period, tiny hydration products were formed on the surface of the raw powder particles. As the hydration process continued, particle clearances were filled with the newly formed hydration products, which shortened the distance between particles and formed chemical bond connection. The existence of the chemical bond reduced the plasticity of pastes and also restricted its flow, leading to the set of Sr-CPC. From this viewpoint, the amount of hydration products actually determined the setting time of Sr-CPC. Table 4 shows that under the same preparation conditions, the setting time of Sr-CPC is prolonged with the increase of Sr content. It could be inferred that the incorporation of Sr ions has an inhibition effect on the formation of hydration products, which is also consistent with the above results of compressive strength test. Actually, the similar results had also been obtained in many other studies on Sr-CPC bone cement as well (Guo et al., 2005a; Wang et al., 2007; Alkhraisat et al., 2008b; Panzavolta et al., 2008; Yu et al., 2009; Pina et al., 2010; Schumacher et al., 2013; D’Onofrio et al., 2016; Singh et al., 2016; Zhu et al., 2017; Wu et al., 2019). In addition, with the increase of L.C. and the decrease of P/L ratio to some extent, the setting time of the cement obviously shortened. It is easily understood that the increase of L.C. enhanced the transformation rate of Sr-HAP, in decent agreement with the above results of compression strength. Previous studies (Gong et al., 2007) suggested that excess liquid in the CPC system could enlarge the distance between particles in raw materials, so that the paste was not compact. Meanwhile, the hydration products generated in early hydration process were unable to contact with each other, which slowed down the setting process. On the contrary, reduced liquid was unable to completely moisten the powder surface so that particle-like agglomeration appeared, leading to a shortened setting process. Overall, this novel TTCP/Sr-α-TCP cement had a shorter setting time compared with the existing triphase TTCP/DCPA/DSPA (Guo et al., 2005a) and binary TTCP/Sr-β-TCP cement system (Zhu et al., 2017) and would be more conducive to clinical practice.

The TTCP/Sr-α-TCP cement system demonstrated a different hydration process. From the results of the XRD analysis shown in Figure 6, it can be inferred that TTCP, as an alkaline component, would first react with PA and then form the DCPD as the initial hydration products. As the hydration reaction continued, the remaining TTCP reacted with the initially formed DCPD and transformed into HAP. After that, TTCP reacted with Sr-α-TCP and the product was Sr-HAP. HAP, as an intermediate product, was gradually transformed into Sr-HAP after exposed in Sr-contained paste *via* ion exchange. As shown in the XRD pattern, the diffraction peaks of TTCP did not disappeared until 10 h. Finally, Sr-α-TCP had almost entirely transformed into Sr-HAP and the diffraction peaks of Sr-α-TCP became difficult to be identified after 24 h. The final hydration product was Sr-HAP and no other phase was detected. The interrelated reactions can be described as follows (Lerous and Lacout, 2001):

(1) Ca_4_(PO_4_)_2_O + 2H_3_PO_4_ + 7H_2_O → 4CaHPO_4_⋅2H_2_O

(2) 2Ca_4_(PO_4_)_2_O + 2CaHPO_4_⋅2H_2_O → Ca_10_(PO_4_)_6_(OH)_2_

(3) Ca_10_(PO_4_)_6_(OH)_2_ + ySr^2+^ → Ca_10__–_*_*y*_*Sr*_*y*_*(PO_4_)_6_(OH)_2_ + yCa^2+^

(4) 2α-Sr*_*x*_*Ca_3__–_*_*x*_*(PO_4_)_2_ + Ca_4_(PO_4_)_2_O + H_2_O → Ca_10__–__2_*_*x*_*Sr_2_*_*x*_*(PO_4_)_6_(OH)_2_

(5) 3α-Sr*_*x*_*Ca_3__–_*_*x*_*(PO_4_)_2_ + H_2_O → Ca_10__–__2_*_*x*_*Sr_2_*_*x*_*(PO_4_)_6_(OH)_2_

Combining the above hydration reactions and the XRD results shown in Figure 6, it is not difficult to understand the compressive strength variation curve shown in Figure 8. The compressive strength of Sr-CPC almost linearly increased to 11.0 MPa at 2 h, attributing to the formation of a small amount initial hydration product (DCPD). During the time from 2 to 10 h, the compression strength rapidly increased and reached a relative balance at 10 h, which is because TTCP reacted with DCPD and formed HAP. In the later stage (10∼24 h), the compression strength was basically stable at about 17.0 MPa, which related to the slow self-setting process of Sr-α-TCP. After that (from 24 to 72 h), the hydration process reached dynamic equilibrium with continuously dissolving and recrystallizing. At 72 h, the hydration was sufficient and the strength reached the maximum (24.5 MPa).

Based on the report elsewhere (Bruckner et al., 2016), the hydration reaction of CPC can be divided into three stages: dissolution of reactive materials, nucleation and growth of HAP crystals, which is a dissolution-precipitation process. In the early hydration process of Sr-CPC, the powder phase gradually dissolves and releases a large number of Ca^2+^, Sr^2+^, and PO_4_^3–^ to form a supersaturated solution, and the nucleation and growth rate was controlled by the surface dissolution of raw materials (Brown and Fulmer, 1991). During the first 10 h of hydration, the reaction kinetics was controlled by the surface dissolution of the raw materials and the alkaline TTCP fast hydrated to form HAP, so its compressive strength increased sharply. After 10 h, the hydration product covers the raw reactive materials, and the hydration reaction kinetics was controlled by the diffusion of water molecules through the product layer. As a result, the compressive strength kept no obvious variation. The newly formed HAP crystals grow and intertwine with each other, and the CPC density increases. At the same time, the newly formed HAP will redissolve and reprecipitate in SBF until the dissolution-precipitation reaction reaches a dynamic balance and the physical and chemical properties of CPC tend to be stable gradually (Bohner, 2007).

The strength of a material also relates to its microstructure. As shown in Figures 7A–D, within the first 10 h, the spicules and fine pores in cement paste increased gradually, while the size of large pores reduced gradually. The initial reticular particles connected with each other and then filled fine pores gradually. Besides, the surrounding liquid penetrated into the pores inside CPC, where hydration products will be generated continuously. It led to the gradually decrease of volume and number of pores. After 10 h, the microstructure of the paste had no significant change basically. The structure of the hydration products tended to dense after 72 h.

It is interesting that this TTCP/Sr-α-TCP system formed a different hydration product from other Sr-containing cement systems. After being immersed in SBF for 72 h, the hydration product of TTCP/Sr-α-TCP system is a single-phase Sr-HAP, while that of TTCP/Sr-β-TCP system in the previous research (Zhu et al., 2017) is a mixture of Sr-HAP and the few residual Sr-β-TCP. Moreover, the setting time of TTCP/Sr-α-TCP system is shorter than that of TTCP/Sr-β-TCP system under the same setting conditions. The main reason is probably that the dissolution and the hydration rate of Sr-α-TCP are much faster than that of Sr-β-TCP. Therefore, Sr-α-TCP almost disappeared at 24 h, but Sr-β-TCP remained after immersed in SBF for 72 h. In addition, there is an initial hydration product of DCPD, which is detected at 30 min and disappeared at 2 h. As a result, the compressive strength of TTCP/Sr-β-TCP system grows slowly while that of TTCP/Sr-α-TCP system greatly increases in the very initial stages.

Based on the results of this study, it is clear that Sr-α-TCP is an important kind of Sr-containing salt. The preparation method in this paper can be used to create a new Sr-containing CPC system by the replacing Ca ions with equivalent Sr ions into the crystal lattice. Table 1 lists doses of Sr-CPC bone cements with different compositions and hydration products in the present available literatures. It is obvious that the compositions of triphase or single-phase cement system are more complex in general, so that impurity ions such as NO_3_^–^ and Cl^–^ may inevitably be introduced. By comparison with other Sr-CPC systems in Table 1, the Sr-CPC prepared in this study only consists of TTCP and Sr-α-TCP with the simple cement liquid of diluted PA. Also, the preparation technology of Sr-source salt, Sr-α-TCP, is simple without introducing any harmful impurities into the system. Totally, as compared with the existing Sr-CPC systems listed in Table 1, this TTCP/Sr-α-TCP-PA system is much easier to be controlled on its hydration reaction and also be industrialized based on the lower cost. Meanwhile, this TTCP/Sr-α-TCP system possesses the advantages of both CPC and Sr ions, which turn into the research focus in recent years. In addition, the hydration products of the biphasic Sr-CPC were Sr^2+^ substituted HAP and its *t*_I_ and *t*_F_ were 1.0∼4.0 and 7.0∼16.0 min, respectively. The fast-setting character makes it better to adapt to the clinical requirements. MC3T3-E1 cells maintained favorable cytocompatibility on the surface of Sr-HAP and the cell number expanded with the extension of culture time. Sr had no obvious inhibitory effect on cell proliferation. However, for further extending its prospective clinical application, the strength of this biphasic Sr-CPC is not enough to repair bearing bone defect currently, so one of the next important work is to conduct more research to improve its strength.

Last but not least, to design an excellent Sr-CPC system, the basic strategies summarized from the present study and discussion would be advised: (1) the selected Sr-containing salts should have an excellent biocompatibility and not induce some harmful impurity ions into the final product; (2) Sr^2+^ ions could be definitely incorporated in to HAP crystal lattice by replacing equal molar ratio of Ca^2+^ ions instead of only being absorbed on the surface of HAP crystals; (3) Sr-doped apatite is the most preferable phase for the final hydration product; (4) the cement hardened body should possess enough mechanical strength and suitable setting time based on clinical requirement; (5) both the cement powder composition and liquid formulation are simplified as much as possible, which lessens the cost and difficulty for industrialization for a potential clinical application. Considering of Ca/P ratio and acid-base neutralization, it is a potential strategy to develop a promising binary Sr-CPC system composed of one acidic salt and one basic salt with a simple cement liquid phase. The corresponding hydration process is easily controlled and the product cost is also low.

## Conclusion

In this study, an important Sr-containing salt, Sr-α-TCP, was prepared with a one-step sintering way and a new kind of Sr-CPC materials consisted of Sr-α-TCP and TTCP was developed with a binary TTCP/Sr-α-TCP mixture (the cement powder composition) and a simple diluted PA solution (the cement liquid). In the cement-hardened body, the residual Sr-α-TCP particles did not play an *in situ* reinforcing effect but created some newborn pores after being completely hydrated, which was beneficial to the new bone growth. The hydration process of Sr-CPC mainly covered three stages: initial formation of DCPD (30 min∼1 h); complete hydration of TTCP and the initially formed DCPD (2∼6 h); and self-setting of Sr-α-TCP (6 h∼). With the hydration proceeding, the compressive strength of Sr-CPC increased sharply and then displayed a gentle stage after 6 h. It revealed that the compressive strength of Sr-CPC was closely related to the transformation rate of Sr-HAP. MC3T3-E1 cells maintained a favorable cytocompatibility on the surface of Sr-HAP and the cell number expanded with the extension of culture time. Sr had no obvious inhibitory effect on cell proliferation.

## Data Availability Statement

The original contributions presented in the study are included in the article/supplementary material, further inquiries can be directed to the corresponding author/s.

## Author Contributions

All authors have made substantive intellectual contributions to this study. LS: study concepts, study design, literature research, clinical studies, experimental studies, data analysis, manuscript preparation, and manuscript editing. TL and SY: study concepts, study design, clinical studies, experimental studies, and manuscript editing. MM: study concepts, study design, literature research, clinical studies, experimental studies, data acquisition, and manuscript preparation. DG: study concepts, study design, clinical studies, experimental studies, manuscript editing, manuscript review, and manuscript final version approval.

## Conflict of Interest

The authors declare that the research was conducted in the absence of any commercial or financial relationships that could be construed as a potential conflict of interest.

## Abbreviations

Sr-CPC: strontium-substituted calcium phosphate bone cement
HAP: Ca_10_(PO_4_)_6_(OH)_2_
TTCP: Ca_4_(PO_4_)_2_O
DCPA: CaHPO_4_
DSPA: SrHPO_4_
PA: Diluted H_3_PO_4_ hydrous solution
*t*_I_: initial setting time
*t*_F_: final setting time, α-TCP, α-Ca_3_(PO_4_)_2_
β-TCP: β-Ca_3_(PO_4_)_2_, DCPD, CaHPO_4_ ⋅ 2H_2_O, Sr-β-TCP, β-Ca_3__–_*_*x*_*Sr*_*x*_*(PO_4_)_2_
MCPM: Ca(H_2_PO_4_)_2_ ⋅ H_2_O
ACP: amorphous Ca_3_(PO_4_)_2_
SC: SrCO_3_
CC: CaCO_3_
Sr-α-TCP: α-Ca_3__–_*_*x*_*Sr*_*x*_*(PO_4_)_2_
XRD: X-ray diffraction
P/L: weight ratio of powder to liquid
SBF: simulated body fluids
FESEM: field emission scanning electron microscopy
SEM: scanning electron microscopy
EDS: energy dispersive spectrometer
MTT: 3-(4,5)-Dimethylthiahiazo(-z-y1)-3,5-di-phenytetrazoliumromide
TCPS: tissue culture polystyrene
L.C.: liquid concentration.
